# Eave ribbons treated with the spatial repellent, transfluthrin, can effectively protect against indoor-biting and outdoor-biting malaria mosquitoes

**DOI:** 10.1186/s12936-018-2520-1

**Published:** 2018-10-17

**Authors:** Arnold S. Mmbando, Halfan Ngowo, Alex Limwagu, Masoud Kilalangongono, Khamis Kifungo, Fredros O. Okumu

**Affiliations:** 1 Environmental Health and Ecological Sciences Department, Ifakara Health Institute, Ifakara, Tanzania; 2 School of Public Health, Faculty of Health Sciences, University of the Witwatersrand, Parktown, Republic of South Africa; 3 Institute of Biodiversity, Animal Health and Comparative Medicine, University of Glasgow, Glasgow, UK

## Abstract

**Background:**

Long-lasting insecticide-treated nets and indoor residual spraying protect against indoor-biting and indoor-resting mosquitoes but are largely ineffective for early-biting and outdoor-biting malaria vectors. Complementary tools are, therefore, needed to accelerate control efforts. This paper describes simple hessian ribbons treated with spatial repellents and wrapped around eaves of houses to prevent outdoor-biting and indoor-biting mosquitoes over long periods of time.

**Methods:**

The eave ribbons are 15 cm-wide triple-layered hessian fabrics, in lengths starting 1 m. They can be fitted onto houses using nails, adhesives or Velcro, without completely closing eave-spaces. In 75 experimental nights, untreated ribbons and ribbons treated with 0.02%, 0.2%, 1.5% or 5% transfluthrin emulsion (spatial repellent) were evaluated against blank controls using two experimental huts inside a 202 m^2^ semi-field chamber where 500 laboratory-reared *Anopheles arabiensis* were released nightly. Two volunteers sat outdoors (one/hut) and collected mosquitoes attempting to bite them from 6 p.m. to 10 p.m. (outdoor-biting), then went indoors and slept under bed nets, beside which CDC-light traps collected mosquitoes from 10 p.m. to 6.30 a.m. (indoor-biting). To assess survival, 200 caged mosquitoes were suspended near the huts nightly and monitored for 24 h thereafter. Additionally, field tests were done in experimental huts in a rural Tanzanian village to evaluate treated ribbons (1.5% transfluthrin). Here, indoor-biting was assessed using window traps and Prokopack^®^ aspirators, and outdoor-biting assessed using volunteer-occupied double-net traps.

**Results:**

Indoor-biting and outdoor-biting decreased > 99% in huts fitted with eave ribbons having ≥ 0.2% transfluthrin. Even 0.02% transfluthrin-treated ribbons provided 79% protection indoors and 60% outdoors. Untreated ribbons however reduced indoor-biting by only 27% and increased outdoor-biting by 18%, though these were non-significant (P > 0.05). Of all caged mosquitoes exposed near treated huts, 99.5% died within 24 h. In field tests, the ribbons provided 96% protection indoors and 84% outdoors against *An. arabiensis*, plus 42% protection indoors and 40% outdoors against *Anopheles funestus*. Current prototypes cost ~ 7USD/hut, are made of widely-available hessian and require no specialized expertise.

**Conclusion:**

Transfluthrin-treated eave ribbons significantly prevented outdoor-biting and indoor-biting malaria vectors and could potentially complement current tools. The technique is simple, low-cost, highly-scalable and easy-to-use; making it suitable even for poorly-constructed houses and low-income groups.

## Background

Early-biting, outdoor-biting and pyrethroid resistant malaria vectors cannot be adequately controlled using the current primary interventions, namely long-lasting insecticide-treated bed nets (LLINs) and indoor residual spraying (IRS) [1–3]. To address these challenges and accelerate ongoing efforts for malaria control and elimination, complementary tools that are affordable, easy-to-use and scalable are urgently needed [4, 5]. Fortunately, there have been recent advances on various individual products, which can address outdoor-biting, though these still require further assessment and optimization [4]. To maximize benefits, these complementary tools should also be effective at both household and community-level, and readily scalable across multiple socio-economic groups.

A recent review of evidence on proposed complementary vector control interventions identified seven tools for which there is at least some evidence of community-level evaluation of malaria parasite reduction [6]. These included insecticide-treated clothing and blankets, insecticide-treated hammocks, insecticide-treated livestock, larval source management, mosquito-proofed housing, spatial repellents and topical repellents. The authors emphasized that larval source management and topical repellents had the most advanced evidence, but also that the topical repellents were unlikely to offer wide-spread community-level protection [6]. Indeed, personal protection measures with topical repellents or insecticidal clothing may effectively prevent outdoor-bites, but mosquitoes can move from protected to unprotected individuals [7]. These approaches are also affected by poor compliance among users [8], as well as inadequate supply and access.

Spatial repellent products, which protect multiple persons over wide areas, present a viable alternative with minimal diversionary effects [9] while also providing significant community-wide benefits against malaria infections. In one study in Indonesia, where metofluthrin-based coils were provided to households, malaria parasite prevalence was reduced by 52% among users, compared to non-users [10]. Separately, in China, where mosquito coils treated with 0.03% transfluthrin were provided either alone or in combination with LLINs, malaria parasite prevalence was reduced by between 77% and 94% [11]. However, overall evidence remains inconclusive and findings of this China study particularly had very large confidence intervals because of very low number of cases, therefore, reducing the strength of the evidence [11]. Indeed, a recent Cochrane review on this subject concluded that although some studies have found a protective effect, it remains unclear if spatial repellents are effective at reducing the risk of malaria infection, and that further well-designed studies must be conducted in order to improve the certainty of evidence [12].

A major challenge observed in the two trials above was that they both relied on mosquito-coils, which required daily replacement and high user compliance. Fortunately, new formats are now available for dispensing spatial repellents without application on human skin or burning coils, thereby minimizing challenges associated with compliance. For example, in previous studies where transfluthrin was applied to hessian strips and used outdoors, at least 80% bite prevention was observed consistently over 6 months without any sign of mosquito diversion to non-users within an 80 m radius [13]. These area-wide mosquito repellent formats offer protection in form of passively-dispensed vapours, without any external energy for vaporization, and could be highly applicable in low-income or remote communities [14]. With regard to transfluthrin, which is one of the most widely used spatial repellent compounds, hessian-based fabrics have particularly demonstrated a high level of retention for the insecticides, and can maintain efficacy for up to half a year [13, 14]. In east Africa, the transfluthrin-treated hessian is also highly acceptable by the rural communities and can be produced locally, making such approaches even more applicable for low-income rural communities [15].

Another vector control intervention considered readily applicable for low-income households, and which could be highly complementary to LLINs and IRS is improved housing. Despite ongoing economic transitions, millions of people in rural and peri-urban Africa still live in poorly-constructed houses with unscreened windows and open eave spaces. These gaps and spaces let in *Anopheles* mosquitoes, and represent a significant gap in malaria vector control beyond the times when LLINs are effective [16, 17]. Since majority of malaria transmission in Africa still occurs indoors [18], house improvement initiatives, such as screening doors, windows and eave-spaces are among the best for curbing mosquito-borne disease transmission [17, 19]. Indeed, various interventions targeting these spaces already exist. Examples include blocking the eave spaces [19], using insecticidal eave-baffles and window screens [20], and deploying eave-tubes [21]. However, these methods will only target endophilic and endophagic vector populations, leaving the people outdoors exposed to exophagic and exophilic mosquitoes when they are performing various outdoor activities, such as cooking, storytelling and fetching water [22]. Indeed, in many African communities, families spend long evening hours outdoors performing various activities [23, 24], and sometime even entire nights outdoors, due to factors such as high temperatures indoors and small size of houses.

In this current study, a new approach, hereafter called eave ribbons (ER) is presented which: (a) exploits the eave spaces being the preferred entryway for *Anopheles* mosquitoes, (b) improves delivery of spatial repellents such as transfluthrin, (c) does not require frequent retreatments or high-levels of user-compliance, (d) does not restrict human movements, and (e) provides significant protection from indoor-biting to outdoor-biting mosquitoes for potentially long durations without requiring any electricity. This approach is an adaptation from the previously tested transfluthrin-treated hessian fabric [13, 15, 25]. The new format can be easily fitted onto the eave spaces around human houses. Because of its simplicity, the eave ribbon technology provides a readily-scalable option for using effective spatial repellents against common malaria vectors even in poorly-constructed houses in rural and remote communities.

## Methods

### The semi-field environment

**Fig. 1:**
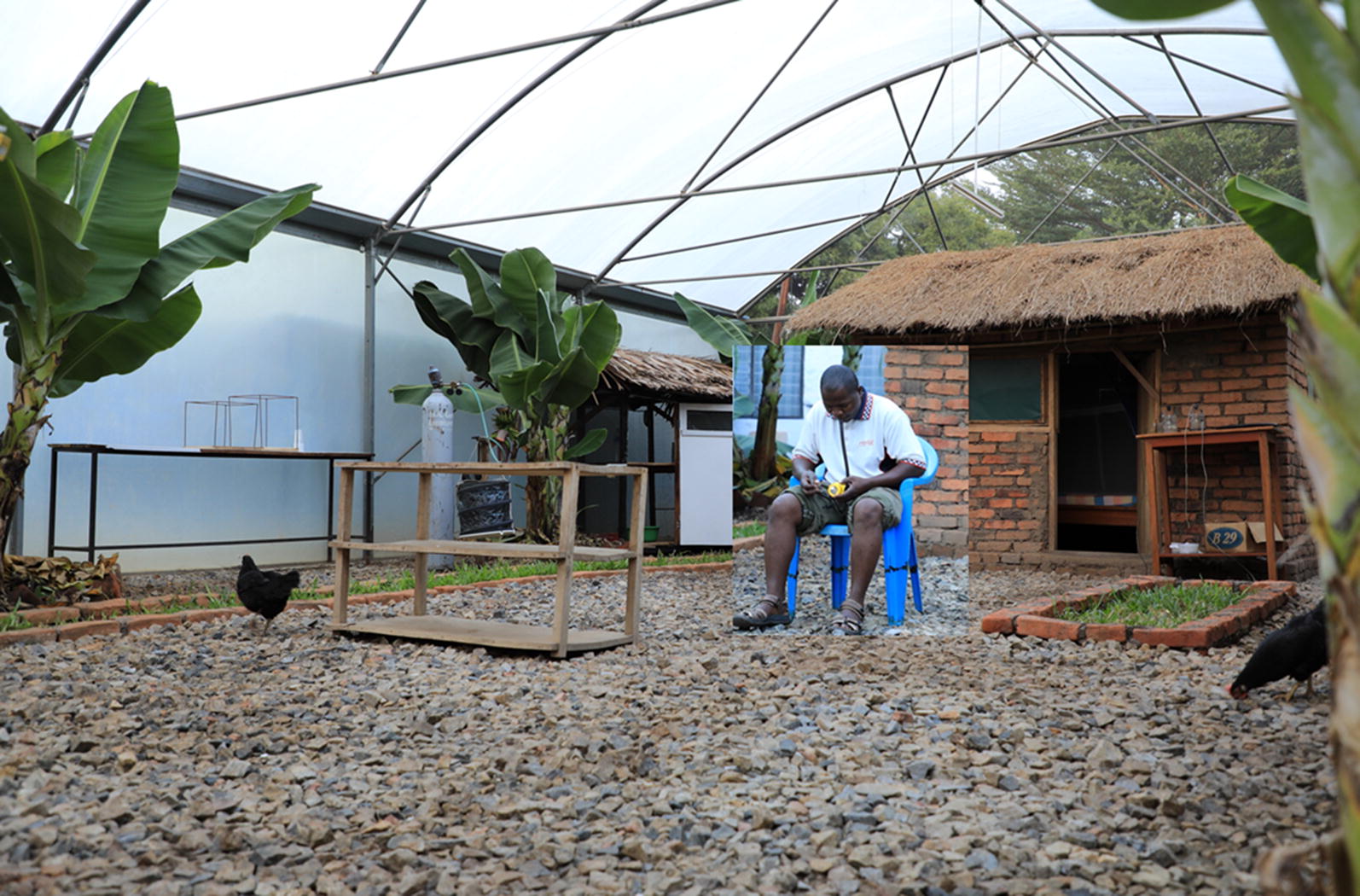
Pictorial illustration of the semi-field chambers and experimental huts used inside the VectorSphere. The semi-field chamber was designed to mimic local mosquito ecosystems in rural villages in Ulanga and Kilombero district, south-eastern Tanzania. There were two experimental huts with brick walls and thatched roofs inside the chamber, which enabled assessment of indoor and outdoor mosquito-biting risk. Each evening, 500 hungry-female *Anopheles arabiensis* mosquitoes were released inside the chamber. Adult male volunteers sat in the peri-domestic space of each of the huts and collected mosquitoes attempting to bite them between 1800 and 2200 h, before going indoors to sleep under intact bed nets. CDC-light traps were used to catch mosquitoes attempting to bite the sleeper between 2200 and 0630 h the next morning. Photograph by Emmanuel Mwanga

The eave ribbons were evaluated in both semi-field and field settings in Tanzania. The semi-field experiments were conducted inside a large screened cage at Ifakara Health Institute, Tanzania, also known as the VectorSphere. This semi-field facility has an area of 625 m^2^, with three separate compartments [26]. The studies here were conducted inside one of the chambers (9.6 m × 21 m), in which vegetation was grown and small livestock (chicken) kept to mimic natural ecosystems. Two experimental huts were constructed 11.5 m apart. The huts were similar to typical local houses in surrounding villages; they had brick walls and grass-thatched roofs, one door and four windows each, and a 20 cm wide eave spaces all round. The huts measured 3.1 m × 2.7 m, and were each fitted with one bed covered with an intact non-insecticidal bed net (Fig. 1).

### Mosquitoes

Five hundred 4–8 days old nulliparous female insectary-reared *An. arabiensis* mosquitoes were released each evening at the centre of the semi-field chamber at 1800 h and left for 30 min before the actual experiment commenced. The mosquito colony had been maintained since 2009, with initial batch of mosquitoes originally from Lupiro village, Ulanga district, Tanzania [27, 28]. The mosquito strain and rearing procedures have been described elsewhere [28]. However, in brief, the rearing conditions included a 12 h:12 h photoperiod, the larvae fed on Tetramin^®^ baby fish food, and emergent adults maintained at temperatures of 27 ± 2 °C and 70–90% relative humidity.

### The transfluthrin-treated eave ribbons

#### Design of the eave ribbons

**Fig. 2:**
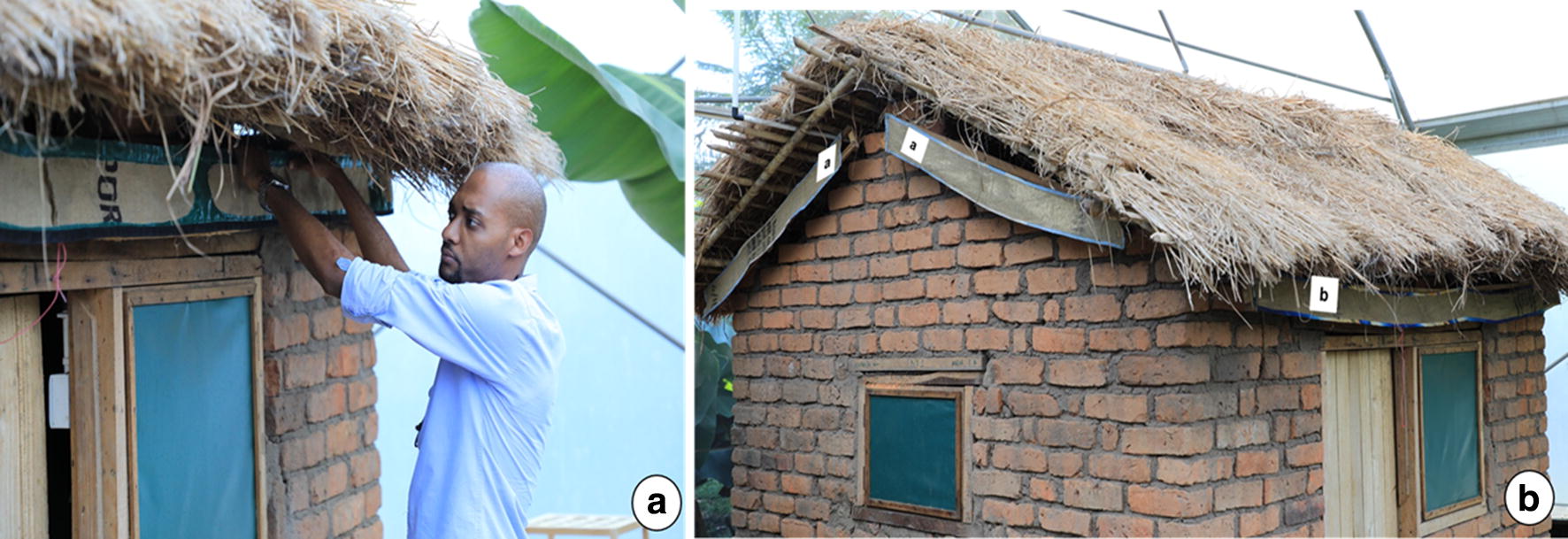
Pictorial illustration of the eave ribbons and their installation along the eave-spaces of the experimental huts (**a**). In the current trials the eave ribbons were designed in lengths of either 1 m or 1.5 m, so multiple pieces were used to cover the entire eave space of the huts (**b**). However, the ribbons could be designed and manufactured with longer lengths, then cut to fit specific house sizes. The ribbons are fitted such that they do not completely cover the eave space, but also without directly touching the experimental hut surfaces, to avoid any contamination (**b**). During the tests, both the two experimental huts in the chamber were either fitted with or not fitted with the eave ribbons. At the end of each set of tests, the chambers were left free for at least 2 days and two nights to prevent residual effects of the treatments. Photographs by Emmanuel Mwanga

The eave ribbons were made of triple-layered hessian fabric woven using sisal fibres. The material is readily-available in Tanzania and is commonly used for manufacturing gunny bags for grain storage. The fabric was cut in multiple sections to fit the perimeter of the experimental huts (Fig. 2). The ribbons used here were either 15 cm wide and 2.5 m long (for fitting onto the front and back sides of the huts) or 15 cm wide and 1 m long (one pair for fitting on the right side and another pair for the left-side of the huts). Since the eave-spaces of the huts were 20 cm wide, the eave ribbons did not fully cover the entire eave-spaces, but instead left gaps of up to 5 cm wide. Indeed, the ribbons were not designed as complete physical barriers against approaching mosquitoes (Fig. 2a, b). Instead they are flexible units that can be fitted around any house design regardless of the design or construction method. The hems of the fabric were tacked and knitted tightly in a canvas cover for ease of handling and to enhance durability.

#### Treatment of the eave ribbons

The eave ribbons were treated with a commonly used spatial repellent, transfluthrin [11, 14, 29]. The ribbons were first washed thoroughly using a liquid detergent, Axion^®^ (Orbit Chemical Industries Ltd, Nairobi) to remove any impurities. After drying, the ribbons were treated with different doses of transfluthrin, following procedures previously described by Ogoma et al. [14, 30]. A technical grade transfluthrin with 97% purity (Shenzhen Sunrising Industry Company^®^, China) was used. To achieve the percentage transfluthrin doses of 5%, 1.5%, 0.2%, 0.02% and 0%, standardized-sized pieces of the ribbons were separately soaked in emulsions containing 50 ml, 15 ml, 2 ml or 0.2 ml, mixed with 50 ml, 85 ml, 98 ml or 99.8 ml of the liquid detergent, respectively, plus 900 ml water in each case; thus, achieving a total volume of 1000 ml. The ribbons were treated in sets, with total surface area of 1.2 m^2^, which included two pieces measuring 1.5 m × 15 cm, and another four pieces measuring 1 m × 15 cm. Given the purity (97%) and density (1.51 g/cm^3^) of active ingredient, and the treatment method used, the final amount of transfluthrin per surface area in the ribbons was 0.25 g/m^2^ w/w (equivalent to 0.02% transfluthrin), 2.47 g/m^2^ w/w (equivalent to 0.2% transfluthrin), 18.50 g/m^2^ w/w (equivalent to 1.5% transfluthrin), and 61.66 g/m^2^ w/w (equivalent to 5% transfluthrin). A separate set of eave ribbons was prepared without any transfluthrin but which had been soaked in a mixture 100 ml of the liquid detergent and 900 ml water and used as the untreated controls.

After treatment, the ribbons were left to dry under shade (away from direct sunlight), then neatly packed in labelled plastic bags. Thereafter, the eave ribbons (treated or untreated) were fitted on eave-spaces of the experimental huts inside the semi-field (Fig. 2) as per the experimental plan described below, but were removed each morning.

### Study procedures

#### Semi-field experiments

Six different experiments were conducted to evaluate the ribbons in the VectorSphere, all lasting a total of 75 test nights plus several nights in between to minimize residual effects. The experiments were conducted between 6 p.m. and 6.30 a.m. each night. A modified before-and-after experimental design was used to evaluate efficacies of the eave ribbons against malaria vector biting risk indoors and outdoors compared to the control settings. For each treatment (i.e. untreated ribbon or ribbons treated with 5%, 1.5%, 0.2% or 0.02% transfluthrin), baseline assessment was first conducted for five consecutive nights with no ribbons fitted to the huts (i.e. controls), and thereafter introduced the intervention and continued the assessment of biting risk for ten consecutive nights. This way, each eave ribbon was tested for a total of 15 nights, with 2 days of no experimentation before testing another concentration, so as to clear any residual effects in the chamber (Table 1). Nightly temperatures and humidity were monitored using Tinytag^®^ data logger (Gemini, UK).

**Table 1:** Summary of semi-field evaluations of eave ribbons. The table shows details of the experiments and also whether the eave ribbons were treated or untreated, as well as the concentration of transfluthrin used. The set ups were as shown in Fig. 3

Experiment	Control (no ribbons)	Untreated ribbons	Transfluthrin-treated eave ribbons				No. nights	Brief description
			5%	1.5%	0.2%	0.02%		
Experiment 1	Yes	n/a	n/a	n/a	n/a	n/a	10	Baseline control experiment to assess outdoor-biting risk (using human landing catch) and indoor-biting risk (using CDC light traps)
Experiment 2.1	Yes	Yes	n/a	n/a	n/a	n/a	15	Evaluating the physical barrier effect of the untreated eave ribbons (the ribbons here had no transfluthrin but were soaked in a mixture of detergent and water). Methods for assessing outdoor-biting and indoor-biting risk were same as in experiment 1
Experiment 2.2	Yes	n/a	Yes	n/a	n/a	n/a	15	Evaluating protective efficacy of eave ribbons treated with different concentrations of transfluthrin, i.e. 5%, 1.5%, 0.2% and 0.02%. Methods for assessing outdoor-biting and indoor-biting risk were same as in experiment 1
Experiment 2.3	Yes	n/a	n/a	Yes	n/a	n/a	15	
Experiment 2.4	Yes	n/a	n/a	n/a	Yes	n/a	15	
Experiment 2.5	Yes	n/a	n/a	n/a	n/a	Yes	15	

#### Set up for assessing indoor and outdoor biting risk

In the control setup, two adult male volunteers (aged 23 and 28 years) sat outdoors to collect mosquitoes attempting to bite them by performing human landing catches (HLC) [31] from 6 p.m. to 10 p.m. each night. After this period, the volunteers entered the huts and slept under intact untreated bed nets until the next morning. This was done to mimic natural behaviours of people in nearby communities, where adults and children often spend time outdoors during early-evening hours before going indoors after 10 p.m. to sleep under the bed net [22, 32]. During the time when volunteers were sleeping indoors, CDC-miniature light traps were used to collect mosquitoes attempting to bite the sleepers from 10 p.m. to 6.30 a.m. the next morning. Using this approach, it was possible to consistently assess outdoor-biting risk (6 p.m. to 10 p.m.) and indoor-biting risk (10 p.m. to 6.30 a.m.) in a standardized way with or without eave ribbons fitted to the huts. The volunteers working in the project remained the same and were fixed to specific huts. This way the volunteer and the hut were consider a single source of experimental variation. To minimize potential sources of variations associated with differential human attractiveness to mosquitoes [33], comparisons were made on a “before-and-after” basis, rather than “between-huts” basis. This arrangement also mimicked the normal leaving condition of people in the village, where individual household members remain fixed at specific houses.

#### Efficacy of transfluthrin-treated eave ribbons on malaria vector biting risks indoors and outdoors

**Fig. 3:**
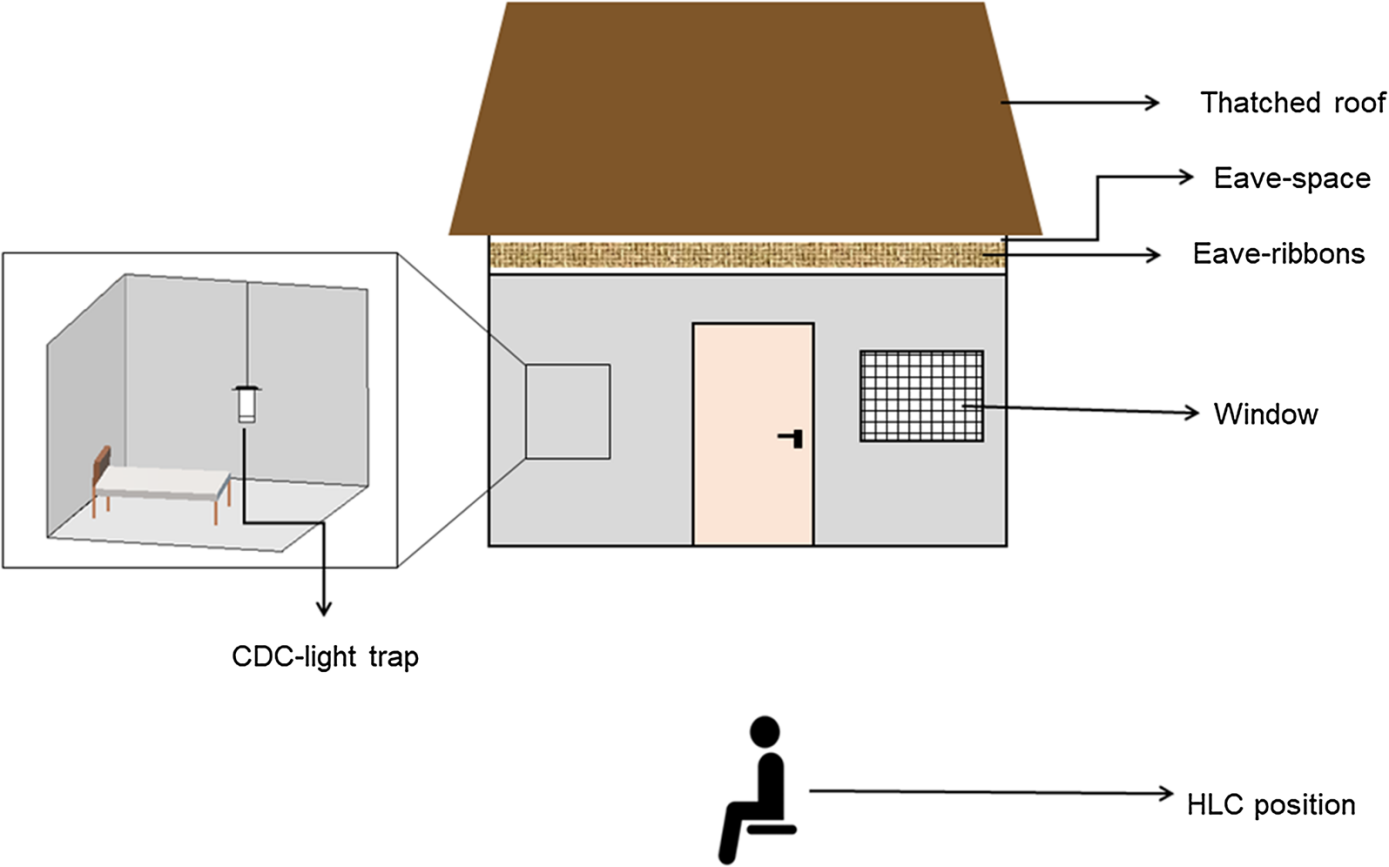
Illustration of the experimental setup used to assess protective efficacy of the transfluthrin-treated eave ribbons inside the semi-field chamber. Two volunteers (one volunteer/hut) conducted HLC outdoors from 6 p.m. to 10 p.m. and thereafter moved inside the huts to sleep under untreated bed-net from 10 p.m. to 6.30 a.m., during which CDC-light traps were used to collect mosquitoes indoors in each hut. In the treatment setup, the huts were fitted with either untreated eave ribbons, or eave ribbons treated with 5%, 1.5%, 0.2% or 0.02% transfluthrin. Each concentration was tested individually for a total of 15 nights, with 2 days of resting before the next concentration was tested. Mosquito collections were done using similar approaches in controls and treatment days

Five different sets of transfluthrin-treated eave ribbons were evaluated over the 75 active test nights. These included, ribbons treated with 5%, 1.5% 0.2% and 0.02% transfluthrin as well as untreated ribbons. Each set of ribbons was tested separately over 15 nights as described above, i.e. for five control nights (no ribbons fitted) and 10 treatment nights (with the eave ribbons fitted). After each 15 nights of testing period for each set, the chamber was thoroughly cleaned for 2 days in order to reduce residual repellents aerosols before introducing another set of treated ribbons (Fig. 2). The actual set of experiments and mosquito trapping stations are shown in (Fig. 3).

In the experiment where the lowest dose of transfluthrin (0.02%) was tested, survival of mosquitoes which were not repelled was assessed over 24 h, i.e. any carry-over effects. To do this, a 15 cm × 15 cm cage containing 100 female *An. arabiensis* mosquitoes was suspended beside each hut each night from start to finish of the experiment. This was replicated 20 times, with a total of 2000 mosquitoes in treatment settings and 2000 in control settings. Mortality of the suspended mosquitoes was evaluated at each morning when retrieving the traps. Where some of the mosquitoes were still alive, the final observation was made after 24 h.

#### Field experiments

Field experiments were conducted in experimental huts [34] located in Lupiro village (8.385°S and 36.670°E) in Ulanga district, south eastern Tanzania, approximately 27 km south of Ifakara town, where Ifakara Health Institute is located. This area has annual rainfall and mean daily temperatures of 1200–1800 mm and 20–32.6 °C, respectively. The major malaria vectors are *An. funestus* and *An. arabiensis* mosquitoes. The main vector control method is LLINs, usually distributed through mass campaigns every 3–4 years and keep-up campaigns done through reproductive health clinics. The vectors are resistant to pyrethroids used in the LLINs, but still mostly susceptible to organophosphates [35, 36].

**Fig. 4:**
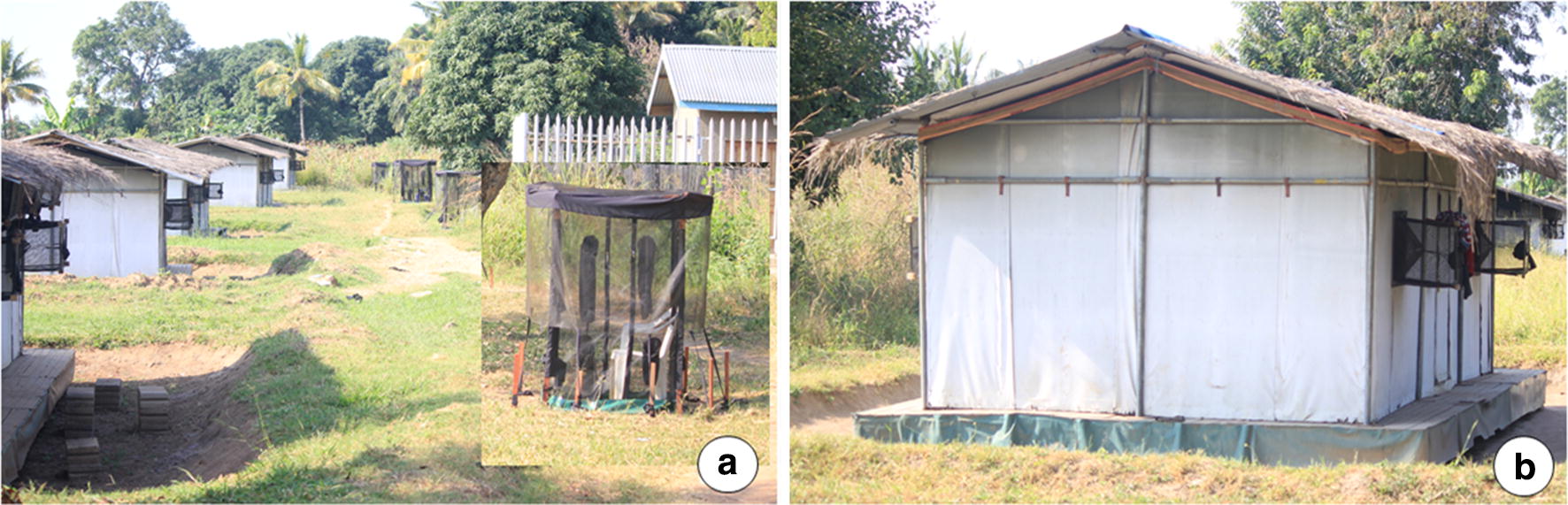
Field evaluation of the protective efficacy of transfluthrin-treated eave ribbons fitted along the eave spaces of experimental huts in rural Tanzania. The figure shows experimental huts fitted with window traps to collect mosquitoes that enter the huts (**a**), and the eave ribbons which were fitted along the eave spaces all around the huts without completely closing off the eaves (**b**). The miniaturized double net traps used to catch host-seeking mosquitoes outdoors but near the huts are also shown (**a**)

The experimental huts used here have previously been described in greater detail elsewhere [34]. In brief, they are built to match the average house size in the local villages and are roofed with iron sheets overlaid with grass-thatch to regulate temperatures. The outer walls are made of canvas, while inner walls are made of cardboard. The huts have eave spaces all round, four windows each and one door. In this study, the windows were fitted with window exit traps to catch mosquitoes that had entered the huts (Fig. 4a).

Eight experimental huts were used concurrently. Four of the huts were fitted with eave ribbons treated with 1.5% transfluthrin solution and the other four huts left without ribbons, to act as controls. The ribbon allocation was swapped weekly over a 4-week experimental period, such that at the end of the study, each hut had been fitted with the ribbons for 2 weeks and used as control also for 2 weeks. At the end of each week, during which only four nights was used to conduct the experiment s, then the huts were left free for two nights, to remove any residual effect of the treatments after the swapping. In the field tests, the untreated ribbons were not evaluated since these had been fully evaluated in semi-field (Fig. 4b). Instead, only treated ribbons were tested and the controls consisted of no ribbons at all. The decision to use 1.5% transfluthrin treatments was based on assumption that field environmental conditions would lead to faster decay of efficacy and also that the mosquitoes would be less susceptible. Eave-ribbons treated with 1.5% transfluthrin, a dose which had offered > 99% protection indoors and outdoors in the semi-field, was therefore used for field evaluation. This field experiment lasted 16 test nights in total.

Mosquito collection in the experimental huts was done as follows: eight adult male volunteers occupied the experimental huts each night (one volunteer/hut; four in treatment huts and four in control huts) and slept under intact LLINs. At the peri-domestic area outdoors, there were another eight human volunteers occupying miniaturized double net traps (one volunteer/trap; four beside the treatment huts and four beside the control huts), which allowed them to safely collect host-seeking mosquitoes without being bitten.

Each night starting 6 p.m. to 6.30 a.m. the next morning, the volunteers sat outdoors, 5 m from the huts inside a mini double net trap designed to allow exposure-free collection of host-seeking mosquitoes. The mini double net (also called Double Net Mini, or DN-Mini) is a modified version of the double net trap previously used for sampling outdoor-biting mosquitoes in south-east Asia [37], but was in this case miniaturized to fit just one sitting volunteer and to make it more portable. Full evaluation of this approach for mosquito sampling has been described elsewhere (Limwagu et al. unpublished). Using this approach, the outdoor-biting risk near the experimental huts was estimated for the entire night. The volunteers stayed fixed at each hut but interchanged each night between indoor and outdoor stations; i.e. being either inside the hut sleeping under LLIN or outside in the mini-double net trap.

The indoor biting risk was assessed using a combination of exit window trap catches. Also, resting mosquitoes were collected once each morning using a Prokopack^®^ aspirator. Similar procedures were employed in huts fitted with eave ribbons and control huts. The experimental set up is shown in (Fig. 4a, b).

### Data analysis

Data analysis was done using open source statistical software, R version 3.3.2 [36], using *lme4* [38], *ggplot2* [39] and *dplyr* [40] packages. Mosquito count data were modelled using generalized linear mixed model (*glmm*) following a negative binomial distribution to account for overdispersion. Number of mosquitoes caught in both semi-and field experiments were included in the model as a response variable while interventions were included as fixed factors. Experimental hut ID and day were included as random terms to account for any variation in microclimatic condition between days. The intervention side included control (no ribbon), untreated ribbons, treated ribbons of different transfluthrin (5%, 1.5%,0.2% and 0.02%). Model coefficients were exponentiated to obtain relative rates of catching mosquitoes inn the respective huts (RR) and the respective 95% confidence intervals. Data obtained from the experimental hut trials (i.e. from window traps, double net traps and Prokopack^®^ aspirators) in the field was analysed the same way using GLMMs and *lme4* package in R. It was fitted to negative binomial distribution with log-link functions to correct for overdispersion. The treatments or control labels were used as fixed factors and experimental day and hut id used as random factors in the analysis. Percentage protective efficacy were calculated using the adjusted means from a *glmm* models with no intercept with the formula $\frac{Control - Treatment}{Control}*100$. The graphs were created using ggplot package [39]. The significance level was considered when P-values is less than 0.05.

## Results

### Semi-field tests

**Fig. 5:**
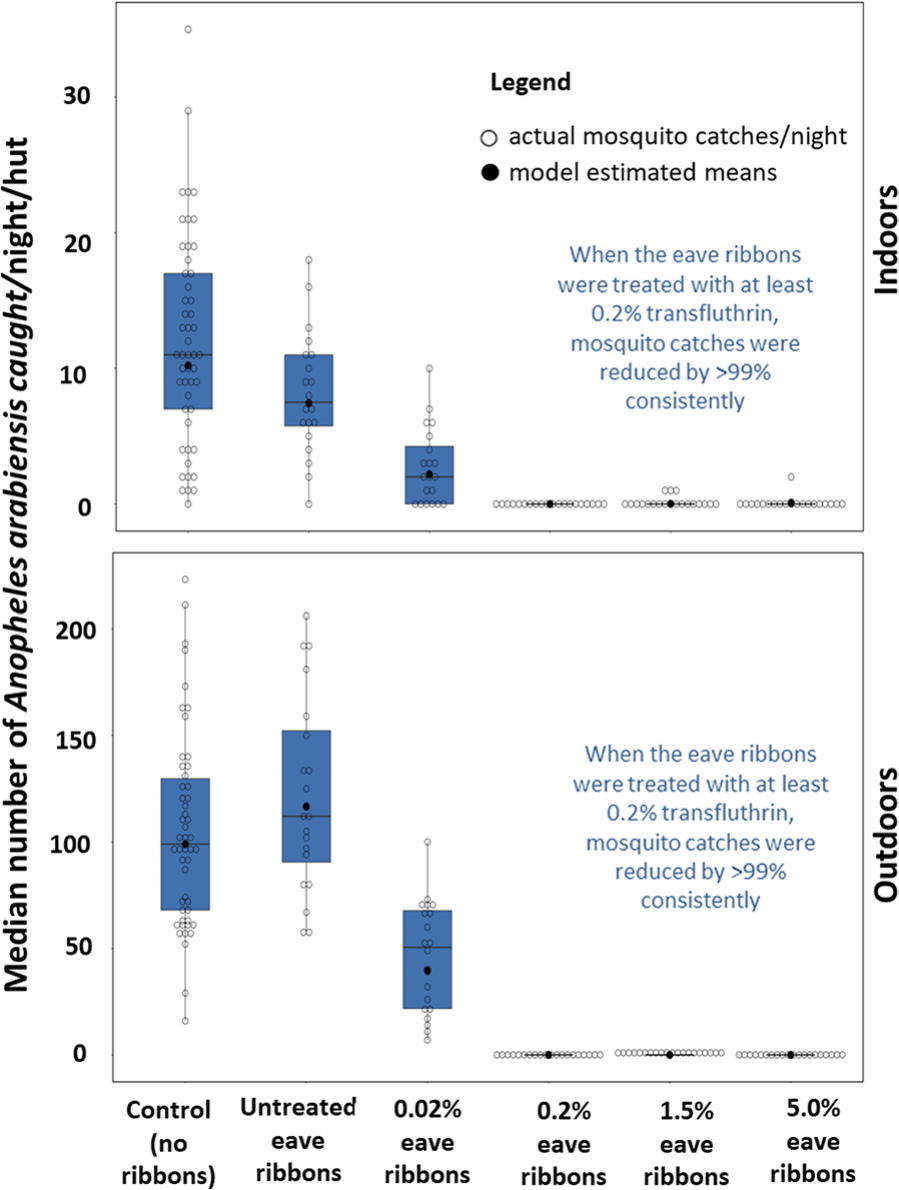
Median number of *Anopheles arabiensis* mosquitoes caught per hut per night outdoors (by human landing catches) and indoors (by CDC light traps), when the huts had either no eave ribbons fitted (controls) or were fitted with untreated or treated eave ribbons. The figure shows both the actual mosquito counts per night and the model estimated mean catches

Nightly indoor-biting and outdoor-biting rates decreased by more than 99% in huts fitted with eave ribbons having at least 0.2% transfluthrin. Results for the evaluation of untreated or treated eave ribbons are illustrated in Fig. 5, Table 2. Eave ribbons treated with 5% transfluthrin reduced number of mosquitoes caught in CDC light traps indoors by 99.2% (95% CI 99.00–100%) and number attempting to bite volunteers outdoors by 100%, compared to controls. Similar protection levels were observed with eave ribbons treated using 1.5% transfluthrin (99.9% reduction in mosquito catches indoors and outdoors). Similarly, eave ribbons treated with just 0.2% transfluthrin solution also provided complete protection indoors and outdoors (100% reduction in mosquito catches by both CDC-light traps and HLC). Even with the lowest dose, i.e. 0.02% transfluthrin-treated ribbons, there was still significant levels of protection indoors and outdoors compared to controls. These sets of ribbons reduced the catches indoors by 77.2% (94.12–99.68%) and catches outdoors by 56.2% (31.43–76.5%), compared to controls. The untreated eave ribbons however prevented only about one-third of mosquitoes from entering the huts, but slightly increased the biting risk outdoors. Compared to the controls, the number of mosquitoes caught indoors in huts with untreated eave ribbons was reduced by 32% (28.9–63.7%), while the number caught outdoors was increased by 16% (− 61–17.6%). These effects of untreated eave ribbons were however not statistically significant relative to controls (P > 0.05).

**Table 2:** Mean number of *Anopheles arabiensis* mosquitoes caught indoors and outdoors when different transfluthrin-treated eave ribbons were fitted to the experimental huts inside the semi-field chamber

Intervention	N	Indoor-biting risk (assessed using CDC-light traps)			Outdoor-biting risk (assessed using human landing catches)		
		Mean [95% CI]	% Protection	*P*-value	Mean [95% CI]	% Protection	P-value
Control	25	12.06 [7.85–16.27]	Reference		105.20 [80.3–130.06]	Reference	
Untreated eave-ribbons	10	8.15 [4.25–12.05]	32.4%	> 0.05	121.80 [82.42–161.18]	− 15.7%	> 0.05
Eave-ribbons 5%***	10	0.10 [0.00–0.48]	99.2%	< 0.001	0.00 [0.00–0.00]	100.0%	< 0.001
Eave-ribbons 1.5%***	10	0.01 [0.00–0.47]	99.9%	< 0.001	0.15 [0.00–0.57]	99.9%	< 0.001
Eave-ribbons 0.2%***	10	0.00 [0.00–0.00]	100.0%	< 0.001	0.00 [0.00–0.00]	100.0%	< 0.001
Eave-ribbons 0.02%***	10	2.75 [0.32–5.18]	77.2%	< 0.001	46.10 [23.5–68.57]	56.2%	< 0.001

N refers to number of experimental nights (N values for the control are summed up across the experiments and baseline data omitted). The mean nightly mosquito catches are estimated using GLMMs at 95% confidence intervals (CI). The % Protection refers to percentage reduction in catches relative to controls

***Values refer to percentage of transfluthrin used to treat the eave ribbons

The possibility of mosquito diversion was not assessed in this study. However, in the tests where caged female *An. arabiensis* mosquitoes were exposed inside huts fitted with eave ribbons having 0.02% transfluthrin, up to 99.5% (1990/2000) mortality was observed compared to 16% (320/2000) in control settings.

The mean nightly temperature recorded inside the SFS-chamber by using the Tinytag^®^ data logger during the rainy seasons was 22.3 °C [21.9–23.2 °C] and the RH (also measured by Tinytag^®^) was 63.3% [60.2–67%]. In dry season the mean nightly temperature was 27.8 °C [26.4–30.5 °C] and the RH was 84.5% [80.6–99.6%].

### Results of the field tests on wild mosquitoes

In the field studies conducted in the malaria endemic village of Lupiro, in rural Tanzania, experimental huts fitted with the transfluthrin-treated eave ribbons had significantly lower malaria vector biting risk than huts without the ribbons. Even in peri-domestic areas outdoors, the number of mosquitoes attempting to bit the volunteers, as estimated by the miniaturized double net trap catches, was lower at the treated huts than control huts.

The highest protection, which was 96%, was observed against *An. arabiensis*. There was an average of only 0.54 [0–1.34] mosquitoes of this species caught per night per hut in the window exit traps in treated huts compared to 13.1 [12.1–14.1] in the control huts. Outdoors, the eave-ribbons offered 84% protection for volunteers siting in the double net traps; these volunteers collected only 1.26 [1.13–1.39] *An. arabiensis* mosquitoes nightly compared to 7.95 [7.15–8.75] in control sites. Recent assessments in this area have consistently demonstrated that the *An. gambiae* complex here comprises entirely of *An. arabiensis* mosquitoes, thus no PCR assays were conducted to distinguish these sibling species). The effects on *An. funestus* was however modest and lower than for *An. arabiensis*, reaching only 42%, 2.1 [1.96–2.24] indoors and 40%, 0.60 [0.25–0.59] outdoors. There was however no effect of the eave ribbons on the biting risk of *Culex* mosquito species, and only marginal reductions were observed in the biting risk from other *Anopheles* species, such as *Anopheles coustani*, *Anopheles ziemmani*, *Anopheles welcommei*, *Anopheles pharoensis* and *Anopheles squamosus* (Table 3). During the experimental period, the mean nightly temperatures were 22.9 °C (19.5–25.7) while the mean relative humidity was 73.1% (68.9–83.7).

**Table 3:** Results of experimental hut evaluation of the protective efficacy of the transfluthrin treated *eave ribbons* fitted along the eave-spaces of the huts

Mosquito species	Nights	Micro-climatic conditions**		Intervention	Indoor mosquito collection				Outdoor mosquito collection	%Pe
		Average Temp (°C)	Average %RH		Window Trap	%Pe	Resting Trap	%Pe	Min-double net-Trap	
					Mean [LCI-UCI] 95%		Mean [LCI-UCI] 95%		Mean [LCI-UCI] 95%	
*Anopheles gambiae*	16	22.9	73.1	Control	13.11 [12.1–14.1]	Ref	0.23 [0.1–0.4]	Ref	7.95 [7.2–8.8]	Ref
	16	22.9	73.1	Treatment	0.54 [0.0–1.3]	96%	0.14 [0.0–0.3]	39%	1.26 [1.1–1.4]	84%
*Anopheles funestus*	16	22.9	73.1	Control	3.6 [3.4–3.8]	Ref	0.47 [0.4–0.5]	Ref	1.0 [0.2–0.7]	Ref
	16	22.9	73.1	Treatment	2.1 [2.0–2.2]	42%	0.16 [0.1–0.3]	66%	0.60 [0.3–0.6]	40%
*Other Anopheles species##*	16	22.9	73.1	Control	0.7 [0.1–0.2]	Ref	0.03 [0.0–0.1]	Ref	3.33 [3.3–3.4]	Ref
	16	22.9	73.1	Treatment	0.27 [0.0–0.2]	61%	0.06 [0.0–0.2]	− 10%	1.50 [1.4–1.6]	55%
*Culex species*	16	22.9	73.1	Control	73.8 [71.5–76.1]	Ref	7.08 [5.7–8.5]	Ref	30.6 [28.9–32.3]	Ref
	16	22.9	73.1	Treatment	51.3 [49.3–53.3]	30%	5.73 [4.7–6.8]	19%	19.23 [18.3–20.3]	37%

Assessment of biting risk was conducted outdoors using human-occupied double net traps and indoors using both exit window traps and Prokopack^®^ Aspirator

** represents the average temperature and relative humidity in the huts which were averaged over the entire duration of the study

## represent other *Anopheles *species caught during the study which are *Anopheles coustani*, *Anopheles pharoensis*, *Anopheles welcommei* and *Anopheles squamosus*

## Discussion

Spatial repellents are considered potential alternatives for malaria vector control and could be applicable alongside existing interventions such as LLINs and IRS. However, there have been challenges associated with low compliance rates [8], poor delivery formats that cannot be readily scaled-up, high costs and lack of effective spatial repellent compounds with suitable safety profiles. The work presented here was an attempt to address most of these challenges by developing a low-cost, easy-to-use and highly scalable format that is applicable for even poorly-constructed houses in remote communities. A format previously tested by Ogoma et al. and demonstrated to provide long-lasting protection up to 6 months [14] or more [13] was adopted. Using the same hessian fabric, simple eave ribbons were created that can be fitted alongside any house type without necessarily covering eave spaces.

This study has demonstrated that the approach can confer protection against indoor and outdoor bites of the major malaria vector *An. arabiensis* in both semi-field and field settings. Targeting the eaves with treated ribbons reduced indoor mosquitoes substantially while also protecting individuals outdoors in the peri-domestic area. This means the technology could be highly suitable for communities where people spend significant amounts of time outdoors before eventually going indoors to sleep under their bed nets. Previous studies targeting eaves with insecticidal treatments were able to demonstrate reduction of indoor densities and biting risks, but did not show any benefits against outdoor densities or biting risk [20]. Similarly, eave screening measures have been widely used to limit malaria vector densities indoors in multiple countries and even demonstrated to reduce malaria incidence [17, 41, 42]. The approach developed here uses spatial repellent treated fabrics along the eaves of houses, thereby preventing entry through repellency, while also providing protection to people in the nearby environment. Other additional advantages here would be that: (a) it protects multiple people at the same time, (b) it does not require direct application of the repellents on human skin and (c) by hanging the products high up close to the eaves, it prevents human contact with the treated surfaces.

A major determinant of the overall efficacy of this approach was the concentration of transfluthrin used. While this study was primarily designed to demonstrate potential of this approach of using eave ribbons, it will be important that future developments of the technology focus also on finding appropriate active ingredients and doses that are both effective and safe. In this study various dosses were assessed and observed high levels of protection even with doses as low as 0.02% transfluthrin, equivalent to 0.25 g/m^2^. Eave ribbons treated with 5%, 1.5% and 0.2% transfluthrin all achieved near complete protection, i.e. > 99% against both indoor and outdoor biting mosquitoes, while the ones treated with 0.02% provided between 56 and 72% protection in the semi-field. These results corroborate the previous studies, which demonstrated that treated hessian strips can offer more than 75% protection against outdoor mosquito bites for long-periods [13].

With regard to indoor mosquito bite prevention, the current study findings also match the previous work which involved window-screening and eave-baffles treated with combination of insecticides, and which also offered significant biting protections indoors [20]. However in those previous studies, and also in other experiments to evaluate related technologies such as eave tubes [21], no effects outdoors were expected and were therefore not measured. In this current study however, the untreated eave ribbons installed along the eave-spaces of the huts did not reduce mosquito densities indoors by more than one-third, and only marginally increased outdoor biting risk, though neither of these effects were statistically significant relative to controls. This demonstrates that biting prevention offered by the ribbons was due primarily to the spatial repellent treatment as opposed to the physical barrier effect.

Whereas it may be more directly beneficial to just fully screen the houses, the eave ribbons approach enables protection for even poorly-constructed houses with multiple other openings on walls and eaves, but which can still be protected without full screening. This way, the technology is more readily scalable even to very low income households and even in housing structures considered not amenable to screening or other technologies such as eave tubes [21] or eave baffles [20]. In an ongoing study in Tanzania, the technology is currently being evaluated for protection of migratory rice farmers who typically dwell in temporary semi-open shanty-like structures for long period of time (sometime up to 6 months) while tending to their crops (Kyeba Johnson Swai; Personal Communication). While house screening, IRS or LLINS may not be readily applicable for such migratory farming households [32] the eave ribbons approach would be directly applicable. The technology could potentially also be applicable to other itinerant populations e.g. pastoralists, fishermen and forest workers.

Both personal and household protections offered by the eave ribbons are crucial not only in south-eastern Tanzania, but more generally in context of community life in many rural malaria-endemic developing countries. In such settings, early in the evening and mornings, significant proportions of individuals are usually active within the peri-domestic area, performing various activities e.g. cooking, storytelling, washing dishes and performing other domestic activities that put them at risk of being bitten by disease transmitting mosquitoes if not protected [22, 24, 32]. It can be expected that technologies such as the one tested here would offer protection to multiple family members outdoors. In the different study when the treated hessian strips were used outdoors, there was sufficient biting protection within a 5 m radius [13], which was also the case for the treated eave ribbons when fitted along the eave-spaces of the hut shown to offer protection to human at peri-domestic areas.

The levels of protective efficacy demonstrated by the treated eave ribbons in this study, could potentially be further improved by adding odour-baited traps or lure and kill technologies so as to achieve high levels of communal level protection beyond the household and personal protection currently observed. Indeed, this has already been demonstrated in small-scale in push–pull approaches [43, 44]. A study by Menger et al. demonstrated that such push–pull effects may however be greater at community level than in the peri-domestic areas [43], most likely because of the traps, when placed near houses lure the mosquitoes to the area, potentially increasing risk in the peridomestic space, and but the trapped mosquitoes are killed, thereby reducing overall risk at community level overtime. It is particularly interesting that the Menger et al. study also applied a form of eave wrappings similar to the eave ribbons used here, though using a different fabric. It is clear therefore that this approach, though originally tested as a component of push–pull could be a highly effective stand-alone product for personal and household level protection (Table 2).

In the field settings the eave-ribbons offered significant protection of more than 80% against *An. arabiensis* mosquito bites for both outdoors and more than 90% indoors. This corroborates results obtained in the semi-field system. Additionally, there was more than 30% reduction in *An. arabiensis* mosquitoes found resting on the walls of the huts with the eave ribbons compared to huts without the ribbons (control). However, for *An. funestus*, also a major malaria vector, the ribbons offered only modest protection of approximately 40% both indoors and outdoors. This too is in line with a previous study, which involved using the transfluthrin actively dispensed at the peri-domestic areas in a push–pull approach whereby the approach did not significantly reduce *An. funestus* biting and it increased possibility of mosquito diversion effect [44]. The unresponsiveness of *An. funestus* towards the eave-ribbons might be due to strong levels of insecticide resistance [36] and possibly the strong anthropophilic tendencies of this species [45]. The eave-ribbons also reduced secondary malaria vectors biting risk by approximately 55% at both indoor and outdoor, which is crucial as these vectors can also play a role in contribute malaria transmission [46]. However, the ribbons offered minimal biting protections of less than 40% from the non-malaria vectors such as *Culex* species, which are mostly nuisance biters but can also transmit other mosquito-borne infections like filaria worms and arboviruses.

Although, the eave-ribbons have demonstrated significant protection in this study, this intervention faces a challenge of low temperatures which hinder the vaporization of the transfluthrin (spatial repellents) hence lowering its efficacy in cold evenings and nights or generally in cold climates. In the field evaluation of the push–pull system, average nightly temperatures of 22.9 °C and 73% relative humidity were recorded, at which there was still substantial protection. It has been shown that conditions significantly below room temperature can reduce the biting protection offered by the treated materials such as sisal strips and sisal decorative materials [13, 15]. The intervention is, therefore, mostly useful for the tropical and sub-tropical countries. However, this temperature effect is not considered a major barrier simply because mosquito-borne illnesses are also more prevalent in hot temperate climates than in cold climates. The need for this technology therefore diminishes with diminishing temperatures, and the fabric will retain the active ingredient until temperatures rise, which would be coincident to the time when biting risk also rises. One limitation with this study was that the exact amount of transfluthrin adsorbed into the hessian fibres was not determined. Future developments of this technology could benefit from microencapsulation techniques and also assessment techniques that measure exact doses in the treatments and also the actual decay rates over time at different temperatures.

For these prototypes tested here, only 7 US dollars was needed to make and install the sets of treated eave-ribbons per experimental hut, possibly accommodating four people. This is just under the cost of bed nets, which cost up to 5 USD (including manufacturing and distribution costs), and can protect a maximum of two people only indoors (with no protection to people who are active outdoor before getting indoors to sleep). Thus, the eave ribbon technology, if developed further could offer an effective, scalable and low-cost complementary tool to be used alongside LLINs and IRS even in low income communities.

## Conclusion

Transfluthrin-treated eave ribbons significantly prevent outdoor-biting in peri-domestic areas and also indoor-biting malaria vectors and could potentially complement current tools. The protection primarily due to the spatial repellent treatments as opposed to the physical barrier. The technique is simple, highly-scalable, easy-to-use and suitable even for poorly-constructed houses, thus applicable across multiple socio-economic groups. Current prototypes cost 7 USD/hut, are made of widely-available hessian and require no specialized expertise. The eave-ribbons do not require high-technology, external energy for vaporization, sealing of the eave spaces, and any imported materials, thus it can be useful to many rural and peri-urban communities in low-income countries like Tanzania. The technology is also applicable to different house designs. It effectively addresses the problem with eave spaces being the preferred mosquito entryway, improves the delivery of spatial repellents such as transfluthrin, does not require frequent retreatments or high-levels of user-compliance and does not restrict human movement, yet it provides significant protection against both indoor-biting and outdoor-biting mosquitoes for potentially long durations without requiring any electric power supply. Further improvements may include the addition of the odour-baited devices to create a stimulo-diversionary approach such as push–pull system which could aid to communal level protections.
